# Female genital mutilation/cutting among girls aged 0–14: evidence from the 2018 Mali Demographic and Health Survey data

**DOI:** 10.1186/s12905-024-02940-4

**Published:** 2024-03-15

**Authors:** Bright Opoku Ahinkorah, Eugene Budu, Abdul-Aziz Seidu, Ebenezer Agbaglo, Collins Adu, Edward Kwabena Ameyaw, Anthony Idowu Ajayi, Sanni Yaya

**Affiliations:** 1 School of Clinical Medicine, University of New South Wales Sydney, Sydney, Australia; 2 REMS Consultancy Services Limited, Sekondi-Takoradi, Western region, Ghana; 3 Korle Bu Teaching Hospital, P.O. Box, 77, Accra, Ghana; 4 Centre for Gender and Advocacy, Takoradi Technical University, P.O. Box 256, Takoradi, Ghana; 5 College of Public Health, Medical and Veterinary Sciences, James Cook University, Townsville, QLD 4811 Australia; 6 Department of English and Communication, The Hong Kong Polytechnic University, Kowloon, Hong Kong; 7 Center for Social Research in Health, University of New South Wales, Sydney, NSW Australia; 8 Institute of Policy Studies and School of Graduate Studies, Lingnan University, Tuen Mun, Hong Kong; 9 L & E Research Consult Ltd, Wa, Upper West Region, Ghana; 10 Sexual Reproductive Maternal Newborn Child and Adolescent Health Unit, African Population and Health Research Center, Manga Close, Off Kirawa Road, Kitisuru, Nairobi 00100 Kenya; 11 School of International Development and Global Studies, University of Ottawa, Ottawa, Canada; 12 The George Institute for Global Health, The University of Oxford, Oxford, UK

**Keywords:** Female genital mutilation/cutting, Daughters, Human rights, Public health, Mali, DHS

## Abstract

**Background:**

Female genital mutilation/cutting (FGM/C) is considered a social norm in many African societies, with varying prevalence among countries. Mali is one of the eight countries with very high prevalence of FGM/C in Africa. This study assessed the individual and contextual factors associated with female FGM/C among girls aged 0–14 years in Mali.

**Methods:**

We obtained data from the 2018 Mali Demographic and Health Survey. The prevalence of FGM/C in girls was presented using percentages while a multilevel binary logistic regression analysis was conducted to assess the predictors of FGM/C and the results were presented using adjusted odds ratios with associated 95% confidence intervals (CIs).

**Results:**

The results indicate that more than half (72.7%, 95% CI = 70.4–74.8) of women in Mali with daughters had at least one daughter who has gone through circumcision. The likelihood of circumcision of girls increased with age, with women aged 45–49 having the highest odds compared to those aged 15–19 (aOR = 17.68, CI = 7.91–31.79). A higher likelihood of FGM/C in daughters was observed among women who never read newspaper/magazine (aOR = 2.22, 95% CI = 1.27–3.89), compared to those who read newspaper/magazine at least once a week. Compared to women who are not circumcised, those who had been circumcised were more likely to have their daughters circumcised (aOR = 53.98, 95% CI = 24.91–117.00).

**Conclusion:**

The study revealed the age of mothers, frequency of reading newspaper/magazine, and circumcision status of mothers, as factors associated with circumcision of girls aged 0–14 in Mali. It is, therefore, imperative for existing interventions and new ones to focus on these factors in order to reduce FGM/C in Mali. This will help Mali to contribute to the global efforts of eliminating all harmful practices, such as child, early and forced marriage and female genital mutilation by 2030.

## Background

Female genital mutilation/cutting (FGM/C) is a public health issue that threatens the health and wellbeing of girls and women all over the world [1]. The World Health Organization (WHO) defines FGM/C as “all procedures that are involved in the partial or total removal of the external female genitalia or other injury to the female genital organs for non-medical reasons” [2]. Although FGM/C is primarily centered in 30 countries across Africa and Middle East [2], it also occurs in the diaspora in Australia, New Zealand, North America, and Europe [3]. With a considerable decline in FGM/C in some parts of the world, it is estimated that about 200 million women and girls living the world over have undergone the practice and about 30 million girls are at risk of being cut before their 15th birthday [2].

FGM/C has been known as a violation of the human rights of women and girls by several international rights organizations [4]. Aside being an infringement on human rights of females, the practice also has negative health implications, as it destroys healthy genital tissues, resulting in severe pain, hemorrhage, infections, urinary and menstrual problems, sexual problems and even deaths [5]. FGM/C is also linked with poor reproductive health outcomes, including increased risk of childbirth complications and newborn deaths [6, 7].

Structural complications of the genitourinary system, post-procedural complications, obstetrical complications, and death are forms of health complications as a result of FGM/C [8]. The adoption of UN resolution A/RES/67/146 (intensifying global efforts for the elimination of FGM/C) on December 2012 by all 194 member states of the General Assembly, which includes the 30 countries that are at the pinnacle of the practice, is an indication of the commitment at the global level to end FGM/C. The WHO also condemns the medicalization or the practice of FGM/C carried out by healthcare providers, specifically nurses and midwives globally [5].

In some African countries, FGM/C is considered as a social norm [9] and the prevalence of the practice varies across countries. Mali is one of the eight countries with very high prevalence of FGM/C in Africa, as 89% of girls and women aged 15 to 49 years had undergone the practice in 2013 [1]. However, 58% of girls and women who have suffered FGM/C are daughters of mothers who oppose it [1].

Previous studies on predictors of FGM/C in sub-Saharan Africa (SSA) have focused generally in Burkina Faso [10], Ethiopia, [11], Nigeria [12] and Senegal [13]. In Mali, studies by Cetorelli et al. [14] and Hayford et al. [15] have examined the trends, policies and community influences of FGM. The gap in these studies is that, both studies used data obtained in 2012–2013 despite the existence of current nationally representative data that can provide current literature on the phenomenon. Again, previous studies have not assessed how individual and contextual factors play a role in the circumcision of girls 0–14 years. In the present study, we aim to add to the existing literature by investigating the individual and contextual factors associated with FGM/C of girls aged 0–14 in Mali. This study is needful because, despite the high prevalence of the practice, coupled with the rapid increase of medicalization of the practice in Mali, it is not clear why the practice still exists in the country, especially among girls aged 0–14 who are the next generation of women. Findings from the study will call for collective efforts to address the phenomenon within a wider framework of policies and interventions that will holistically address all harmful practices and all forms of violence against girls and women. This will help Mali to contribute to global efforts of eliminating all harmful practices, such as child, early and forced marriage and female genital mutilation by 2030.

## Methods

### Study area and data source

The study was conducted in Mali. Mali is a landlocked country in West Africa and it is the eighth-largest country in Africa with a land area of over 1,241,238 square kilometres. The country shares borders in the north with Algeria, in the east with Niger, in the northwest with Mauritania, in the south with Burkina Faso and Cote d’Ivorie and then in the west with Guinea and Senegal. Mali’s population is estimated to be 21.9 million with about 65% of its population estimated to be under the age of 25. The predominant religion in Mali is Islamic religion accounting for above 95% of the population.

Data for the study were obtained from the 2018 Mali Demographic and Health Survey (MDHS). The Individual Recode (IR) file, which contains data on women of reproductive age (15–49) was used for this study. The survey is the sixth version since its commencement in 1987 [16]. The MDHS is part of the Demographic and Health Survey (DHS) Program which seeks to gather data on health indicators such as female genital mutilation, family planning methods, fertility preferences, sexual activity, marriage, and other essential population health measures in low- and middle-income countries. It used a two-stage stratified random sampling design. At the primary stage, 379 Primary Survey Units (UPS)/clusters from urban (104) and rural (275) areas were systematically selected. The second stage involved the selection of households from the predefined clusters [16]. Details of the sampling and selection of research participants are available in the final report accessible via https://dhsprogram.com/what-we-do/survey/survey-display-517.cfm. For the purpose of the study, women were included if they had daughters and had complete cases on all the variables considered for the study (n = 5,665).

### Study variables

#### Outcome variable

‘Circumcision of girls aged 0–14’ was the outcome variable in this study. To derive this variable, women of reproductive age who had daughters were asked how many of their daughter(s) had their genital area “nicked with nothing removed,” “something removed,” or “sewn shut.” [17, 18]. The responses ranged from ‘no daughter’ to 1, 2, 3, 4, 5, 6 and 7 daughters. To provide a binary outcome, women who said none of their daughters went through FGM/C were coded as ‘No = 0’ and those who had at least one daughter circumcised were coded ‘Yes = 1’.

#### Independent variables

Twelve independent variables, consisting of nine individual level factors and three contextual level factors, were considered in this study. Age groups (15–19, 20–24, 25–29, 30–34, 35–39, 40–44, 45–49 years), level of education (no education, primary and secondary or higher), partner’s education (no education, primary, secondary or and not known), employment status (employed or unemployed), marital status (never in union, married, cohabiting, and Widowed/divorced/separated), frequency of reading newspaper/magazine (not at all, less than once a week, at least once a week), frequency of listening to radio (not at all, less than once a week, at least once a week), frequency of watching television (not at all, less than once a week, at least once a week), and circumcision status (circumcised or not circumcised) were the individual level factors. The contextual level factors were wealth index (poorest, poorer, middle, richer, richest), sex of household head (male or female), and place of residence (urban or rural). The selection of these variables was based on their associations with FGM/C in previous studies [10, 17–20].

### Statistical analyses

Stata version 14.2 for Windows was used in analysing the data. First, the prevalence of FGM/C in girls was presented together with a distribution of FGM/C in girls across the individual and contextual level factors. Statistical significance of the association between each of the factors and FGM/C in girls was measured using chi-square test of independence at a p-value of 0.05 (see Table 1). Second, multilevel binary logistic regression analysis was carried out to examine the individual and contextual factors associated with FGM/C. Only variables that were significant in the chi-square test were considered for the multilevel binary logistic regression analysis. In terms of the modelling, four models, comprising the empty model (Model 0), Model I, Model II, and Model III were fitted. Model 0 was fitted to show the variance in FGM/C in girls, which could be attributed to the clustering of the primary sampling units (PSUs) without the explanatory variables. Model I modelled the association between the individual level factors and FGM/C in girls. Model II contained the contextual level factors and FGM/C in girls while Model III modelled the individual and contextual level factors and FGM/C in girls. Model comparison was carried out using the log-likelihood and Akaike’s information criterion (AIC) tests. The highest log-likelihood and the lowest AIC were used to determine the best-fit model [21]. Adjusted odds ratio and associated 95% confidence intervals (CIs) were presented for all the models apart from model 0 (see Table 2). To check for high correlation among the explanatory variables, a test for multicollinearity was carried out using the variance inflation factor (VIF), and the results showed no evidence of high collinearity (Mean VIF = 1.20, Maximum VIF = 1.65, and Minimum VIF = 1.01). Sample weight (v005/1,000,000) and svy command were used to correct for over- and under-sampling, and the complex survey design and generalizability of the findings respectively.

**Table 1:** Socio-demographic characteristics of women and FGM/C in girls in Mali (Weighted, N = 5665)

Variables	Frequency (n)	Percentage (%)	FGM/C in girls	Chi-square/p-value
**Age groups**				167.27/p < 0.001
15–19	178	3.1	44.3	
20–24	655	11.6	57.9	
25–29	1342	23.7	69.3	
30–34	1325	23.4	76.2	
35–39	1167	20.6	78.3	
40–44	650	11.5	81.3	
45–49	348	6.1	79.9	
**Level of education**				3.72/0.156
No education	4361	77.0	72.7	
Primary	595	10.5	73.7	
Secondary+	709	12.5	71.8	
**Partner’s level of education**				23.65/<0.001
No education	4090	72.2	72.9	
Primary	459	8.1	74.1	
Secondary+	816	14.4	72.4	
Not known	300	5.3	67.7	
**Marital status**				23.96/p < 0.001
Never in union	58	1.0	58.5	
Married	5484	96.8	72.8	
Cohabiting	24	0.4	59.7	
Widowed/divorced/separated	98	1.7	74.0	
**Employment status**				344.94/p < 0.001
Unemployed	2018	35.6	63.3	
Employed	3647	64.4	77.8	
**Frequency of reading newspaper/magazine**				8.58/0.014
Not at all	5446	96.1	73.0	
Less than once a week	132	2.3	67.0	
At least once a week	87	1.5	62.5	
**Frequency of listening to radio**				22.98/p < 0.001
Not at all	1764	31.2	69.0	
Less than once a week	1277	22.5	72.7	
At least once a week	2624	46.3	75.1	
**Frequency of watching television**				131.52/p > 0.001
Not at all	2331	41.1	68.3	
Less than once a week	1210	21.4	75.5	
At least once a week	2123	37.5	75.9	
**Circumcision status**				
Not circumcised	581	10.3	9.6	
Circumcised	5084	89.7	79.9	
**Wealth quintile**				58.19/p < 0.001
Poorest	1249	22.0	70.5	
Poorer	1088	19.2	69.7	
Middle	1174	20.7	69.1	
Richer	1142	20.2	78.4	
Richest	1012	17.9	76.2	
**Sex of household head**				21.42/p < 0.001
Male	4982	87.9	72.7	
Female	683	12.1	72.5	
**Place of residence**				11.33/0.001
Urban	1235	21.8	74.4	
Rural	4430	78.2	72.2	

*Source* 2018 Mali Demographic and Health Survey

**Table 2:** Multivariable multilevel logistic regression models on individual and contextual factors associated with circumcision of girls in Mali

Variables	Model 0	Model I aOR(95%CI)	Model II aOR(95%CI)	Model III aOR(95%CI)
**Age groups**				
15–19		Ref		Ref
20–24		2.08^*^ (1.11–3.92)		2.27^*^ (1.20–4.29)
25–29		4.29^***^ (21.35–7.83)		4.61^***^ (2.53–8.38)
30–34		8.00^***^ (4.27-15.00)		8.49^***^ (4.55–15.81)
35–39		10.15^***^ (5.51–18.68)		10.87^***^ (5.92–19.94)
40–44		11.75^***^ (6.01–22.99)		13.68^***^ (7.014–26.67)
45–49		16.52^***^ (7.19–37.94)		17.68^***^ (7.91–31.79)
**Partner’s level of education**				
No education		1.19 (0.83–1.69)		1.24 (0.86–1.79)
Primary		1.09 (0.83–1.70)		1.12 (0.72–1.76)
Secondary+		Ref		Ref
Not Known		0.70 (0.33–1.48)		0.63 (0.29–1.36)
**Marital status**				
Never in union		Ref		Ref
Married		0.89 (0.31–2.51)		0.71 (0.25–2.01)
Cohabiting		0.61 (0.12–3.15)		0.55 (0.11–2.80)
Widowed/divorced/separated		2.78 (0.74–10.47)		3.31 (0.85–12.90)
**Employment status**				
Unemployed		1.02 (0.79–1.32)		1.01 (0.78–1.30)
Employed		Ref		Ref
**Frequency of reading newspaper/magazine**				
Not at all		2.21^**^ (1.26–3.86)		2.22^**^ (1.27–3.89)
Less than once a week		1.03 (0.32–3.35)		1.03 (0.32–3.31)
At least once a week		Ref		Ref
**Frequency of listening to radio**				
Not at all		0.94 (0.68–1.30)		0.96 (0.69–1.34)
Less than once a week		0.91 (0.67–1.24)		0.92 (0.67–1.26)
At least once a week		Ref		Ref
**Frequency of watching television**				
Not at all		0.95 (0.69–1.31)		0.96 (0.69–1.33)
Less than once a week		0.92 (0.67–1.27)		0.90 (0.65–1.26)
At least once a week		Ref		Ref
**Circumcision status**				
Not circumcised		Ref		Ref
Circumcised		48.18^***^ (22.44–103.50)		53.98^***^ (24.91–117.00)
**Wealth quintile**				
Poorest			Ref	Ref
Poorer			0.91 (0.60–1.38)	0.86 (0.55–1.35)
Middle			0.75 (0.49–1.16)	0.77 (0.52–1.14)
Richer			1.31 (0.70–2.45)	1.90 (0.90–3.43)
Richest			0.75 (0.34–1.67)	0.94 (0.38–2.30)
**Sex of household head**				
Male			1.05 (0.73–1.52)	1.38 (0.92–2.08)
Female			Ref	Ref
**Place of residence**				
Urban			Ref	Ref
Rural			1.33 (0.61–2.94)	5.65 (4.05–7.89)
**Random effect result**				
PSU variance (95% CI)	5.51 (4.37–6.94)	5.59 (4.02–7.76)	10.45 (7.62–14.33)	1.24 (0.92–1.66)
ICC	62.6%	62.9%	76.0%	63.2%
Wald χ^2^	Reference	253.54***	10.68***	309.30***
Model fitness				
Log-likelihood	-2774.5439	-22222.012	-26039.722	-22002.182
AIC	5553.088	44488.02	52095.44	44060.36
N	5665	5665	5665	5665

*Source* 2018 Mali Demographic and Health Survey

* p < 0.05, ** p < 0.01, *** p < 0.001

N = Sample size, Ref = Reference category, PSU = Primary Sampling Unit, ICC = Intra-Class Correlation, LR Test = Likelihood ratio Test, AIC = Akaike’s Information Criterion

## Results

### Characteristics of the respondents

Table 1 shows results on the characteristics of the respondents. The majority of the women were aged 25–29 (23.7%), had no formal education (77%), had partners with no formal education (72.2%), and were married (96.8%). Most of the respondents were not working (64.4%), never read newspaper (96.1%), listened to radio at least once a week (46.3%), and never television (41.1%). Most of them were circumcised (89.7%), were poor (41.2%), lived in male-headed households (87.9%) and in rural areas (78.2%).

### Prevalence of FGM/C in girls in Mali

**Fig. 1:**
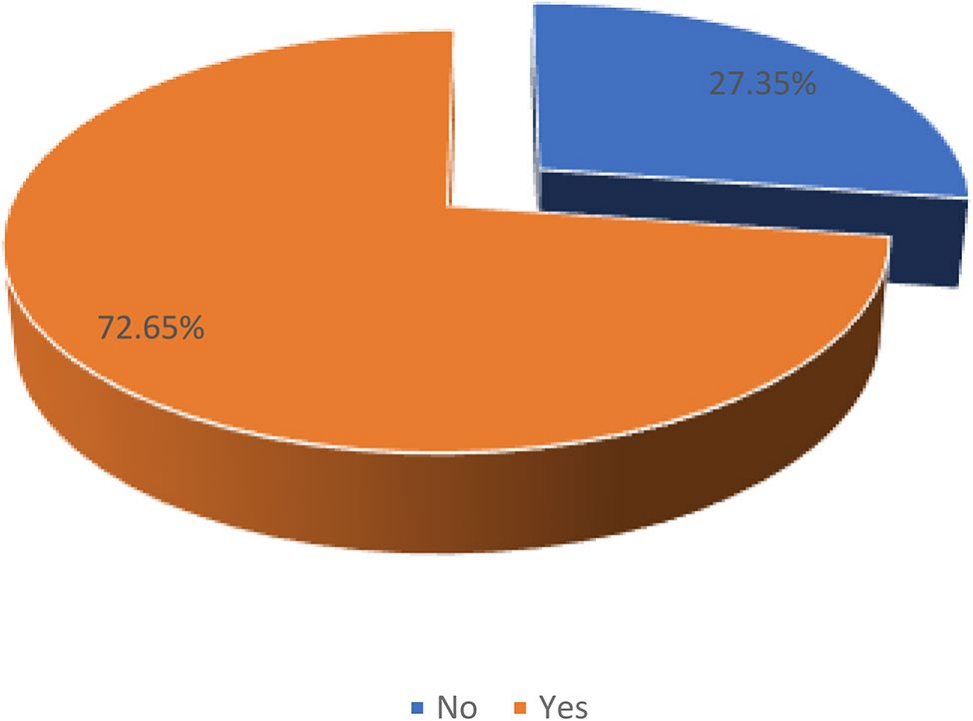
Proportion of women who had at least one daughter circumcised

Figure 1 shows the prevalence of FGM/C in girls in Mali. The results indicate that more than half (72.7%, 95% CI =  70.4–74.8) of women with daughters had at least one daughter who had gone through circumcision.

### Distribution of FGM/C in girls accross the socio-demographic characteristics of women

As shown in Table 1, most of the women whose daughters were circumcised were aged 40–44 (81.3%), had primary education (73.7%), had partners with primary education (74.1%), were widowed/divorced/separated (74.0%), working (77.8%), never read newspaper/magazine (73.0%), listened to radio at least once week (75.1%), and watched television at least once a week (75.9%). Among women who had undergone circumcision themselves, the majority (79.9%) had at least one circumcised daughter, were in the richer wealth quintile (78.4%), lived in male-headed households (72.7%), and lived in urban areas (74.4%). Apart from level of education all the socio-demographic characteristics had significant relationship with the circumcision of girls at 95% CI.

### Predictors of FGM/C in girls in Mali

Table 2 shows results of the multivariable multilevel logistic regression models of the study. As shown in the full model containing all the individual and contextual level factors and circumcision of girls, the likelihood of circumcision of girls increased with age, with women aged 45–49 having the highest odds compared to those aged 15–19 (aOR = 17.68, CI = 7.91–31.79). A high likelihood of FGM/C in daughters was observed among women who never read newspaper/magazine (aOR = 2.22, CI = 1.27–3.89), compared to those who read newspaper at least once a week. Compared to women who had not gone through circumcision, those who had been circumcised were more likely to have their daughters circumcised (aOR = 53.98, CI = 24.91–117.00).

## Discussion

FGM/C negatively affects the health of women, and it is also an infringement on women’s rights. Mali is one of the countries with high rate of FGM/C in SSA. In the present study, we assessed the prevalence and predictors of FGM/C among girls in Mali. The prevalence recorded in this study is 52.5%. This is lower than the 73% reported by one of the organisations in Mali [22] and the 83% reported in the 2012–2013 MDHS [23]. The prevalence of FGM/C in the present study is attributable to a number of factors. Legally, there is no law that proscribes FGM/C in Mali, despite the fact that Mali has endorsed some international resolutions such as the International Day of Zero Tolerance for FGM aimed at fighting FGM/C. This contrasts what happens in other African countries, like Mauritania, where the practice is considered a crime punishable by law [24]. Additionally, in Mali, the practice is buried in social norms, and because of this, people who are willing to stop the practice continue to perpetrate it for the fear of being ostracized by other community members. Thus, many people who might even want to abolish the practice are forced by societal norms to continue the practice [22]. The possible reason for the decrease in the proportion of daughters who were circumcised in Mali between 2013 and 2018 could be attributed to the ongoing education across the globe, especially in Africa, on the negative effects of FGM/C. Again, the reduction in the proportion could be due to our focus on our selection of variables for this study, which limited our analysis to only married and cohabiting women, while the proportion reported in the 2013 MDHS focused on the proportion of all women of reproductive age who reported having their daughter circumcised.

The likelihood of FGM/C was associated with the age of mothers. Specifically, mothers aged 35–39 years had higher odds of getting their daughters circumcised, as opposed to those aged 15–19. Previous studies in Benin City, Nigeria [25], Okada, Edo State, Nigeria [26], Bale zone, Ethiopia [27], and East Gojjam zone, Ethiopia [28] reported similar findings. A study in Iran by Pashael et al. [29] also revealed that older women have positive attitude to FGM/C, relative to younger women. Obi and Igbinadolor [25] explained that the lower likelihood of younger mothers getting their female children circumcised could be as a result of the proliferation of human rights sensitization programs focused on younger women. Such programs normally focus on issues related to negative sociocultural practices including FGM/C. Kaplan et al. [30] also argued that older women normally consider female genital mutilation as a religious practice similar to male circumcision. They, therefore, consider it as a religious obligation, and this could be the reason for the higher likelihood of getting FGM/C done on their daughters. With the lower likelihood of FGM/C among daughters of younger mothers, it is expected that FGM/C will reduce further in the future, if the current trend continues.

The study revealed a significant association between media exposure and FGM/C. Specifically, women who read newspapers or magazines at least once a week had lower odds of getting their daughters circumcised, as compared to those who never read newspapers. This finding disagrees with an earlier study in Mali by Dalal et al. [31], who revealed that teenage girls who read newspapers/magazines had positive attitudes towards FGM/C, compared to those who did not read newspapers/magazines. The difference in findings could be attributed to data source: While Dalal et al. [31] used 2006 Mali Demographic and Health Survey data, which was collected many years, the present study used the 2018 session of the survey. The finding in the present study, compared with what was found by Dalal et al. [31], seems to suggest a change in the influence of media on people’s attitude towards FGM/C over the years. The study used the oldest dataset and this inconsistent finding may indicate that media did not play its expected role of protecting girls from FGM at the time of the study.

Our finding in the present study implies that newspapers and magazines in Mali are now producing contents focusing on FGM/C eradication. In line with this, Cetorelli et al. [24] have noted how some civil society organizations in Mali, including Association Malienne pour le Suivi et l‘Orientation des Pratiques Traditionnelles (AMSOPT), Tagne, Sini Sanuman, are using media such as newspapers to campaign against FGM/C. The low likelihood of FGM/C among daughters of mothers who read newspapers/magazines reported in the present study could be suggestive of the effectiveness of such campaigns. Thus, newspapers and magazines could serve as a medium for health promotion programs aimed at ending FGM/C to reach their audience and clear misconceptions about FGM/C [32].

Also, circumcision status of women had a significant association with getting their daughters circumcised. Specifically, women who had experienced FGM/C were more likely to get their daughters circumcised, compared to those who had not been circumcised. This finding confirms the findings of Obi and Igbinadolor [25], Bogale et al. [33], and Ashimi et al. [34]. This finding suggests that going through FGM/C, with its negative health implications, does not necessarily dissuade women from getting their daughters through FGM/C, as the practice seems to be deeply rooted in some sociocultural norms. Women, therefore, engage in the practice for various reasons [35]. Bogale et al. [33], for instance, identified that FGM/C is done as a religious practice, as a way of safeguarding girls’ virginity, and to ensure one’s social acceptability. Ashimi et al. [34] similarly noted that FGM/C was practiced in order to meet demands of tradition, to enable future husbands of the girls to have pleasure during sex, and to prevent promiscuity. In Mali, FGM/C is supported by some sociocultural and religious norms. For instance, in Dogon religion, the practice is considered as spiritual cleansing [36]. Besides, in Mali, the clitoris is generally associated with masculinity, hence the need to cut it in order to initiate girls into adulthood [22]. This finding suggests that interventions aimed at ending the practice need to focus on eradication of such sociocultural norms and misconceptions.

### Strengths and limitations

It is important to interpret the findings in the light of certain strengths and limitations of the study. In terms of strengths, the data used for the study is nationally representative, and this allows us to generalize the findings to all girls in Mali. Additionally, we used data from the 2018 MDHS, which is the most recent of the surveys. With this, the findings could be reflective of the current practice of FGM/C in Mali. Moreover, we used higher-order statistical tools, such as logistic regressions, for the analysis. This ensured vigorous analysis of the data. Beside these strengths, the study comes with certain limitations. First, due to the age category of girls considered in this study, it is possible that some of the girls would not have been cut at the time of the survey, leading to under-reporting. Again, the study adopted a cross-sectional research design. With this, it becomes impossible to draw causal relationship between the variables studied. Additionally, the retrospective nature of reporting FGM/C may affect the accuracy of the reports, and this may result in recall biases which often characterize DHS data. Relatedly, the reports may also be subjected to issues of social desirability bias. The authors did not control for region of residence due to multicollinearity.

## Conclusion

In the present study, we investigated the determinants of FGM/C in Mali. Firstly, the study revealed an association between the age of mothers and FGM/C of their daughters, with daughters of older women being more inclined to FGM/C. The study also revealed an association between reading of newspapers/magazines and FGM/C. Additionally, the study revealed that mothers who had been circumcised and those who were working were more likely to get their daughters circumcised, compared to those who had not gone through circumcision and those who were not working respectively. It is, therefore, critical for existing interventions and new ones to focus on these factors, so as to reduce FGM/C in Mali. For example, such interventions can successfully use newspapers/and magazines to sensitise women on the need to stop FGM/C in Mali.

## Data Availability

The dataset is available freely for download at: https://dhsprogram.com/data/available-datasets.cfm.

## Abbreviations

cOR: crude Odds Ratio
aOR: Adjusted Odds Ratio
CI: Confidence Interval
DHS: Demographic and Health Survey
FGM/C: Female genital mutilation/cutting
WHO: World Health Organization
Ref: Reference category
PSU: Primary Sampling Unit
ICC: Intra-Class Correlation
LR Test: Likelihood ratio Test
AIC: Akaike’s Information Criterion
